# Abnormal directed connectivity of resting state networks in focal epilepsy

**DOI:** 10.1016/j.nicl.2020.102336

**Published:** 2020-07-06

**Authors:** Margherita Carboni, Pia De Stefano, Bernd J. Vorderwülbecke, Sebastien Tourbier, Emeline Mullier, Maria Rubega, Shahan Momjian, Karl Schaller, Patric Hagmann, Margitta Seeck, Christoph M. Michel, Pieter van Mierlo, Serge Vulliemoz

**Affiliations:** a EEG and Epilepsy Unit, Clinical Neuroscience Department, University Hospital and Faculty of Medicine of Geneva, Geneva, Switzerland; b Functional Brain Mapping Lab, Department of Fundamental Neurosciences, University of Geneva, Geneva, Switzerland; c Epilepsy-Center Berlin-Brandenburg, Department of Neurology, Charité – Universitätsmedizin Berlin, Berlin, Germany; d Connectomics Lab, Department of Radiology, University Hospital of Lausanne, Lausanne, Switzerland; e Department of Neurosciences, University of Padova, Padova, Italy; f Department of Neurosurgery, University Hospital and Faculty of Medicine of Geneva, Geneva, Switzerland; g Medical Image and Signal Processing Group, Department of Electronics and Information Systems, Ghent University, Ghent, Belgium

**Keywords:** ESI, Electrical Source Imaging, iPDC, Information Partial Directed Coherence, LAURA, Local AUtoRegressive Average, LSMAC, Locally Spherical Model with Anatomical Constraints, ROIs, Regions of interest, TLE, Temporal lobe epilepsy, tv-MVAR, Time-varying-multivariate autoregressive, ETLE, Extra-temporal lobe epilepsy, IED, Interictal epileptic discharges, GE, Global Efficiency, RSN, Resting State Network, Epilepsy, Resting State, Connectivity, Network integration, Global Efficiency

## Abstract

**Objective:**

Epilepsy diagnosis can be difficult in the absence of interictal epileptic discharges (IED) on scalp EEG. We used high-density EEG to measure connectivity in large‐scale functional networks of patients with focal epilepsy (Temporal and Extratemporal Lobe Epilepsy, TLE and ETLE) and tested for network alterations during resting wakefulness without IEDs, compared to healthy controls. We measured global efficiency as a marker of integration within networks.

**Methods:**

We analysed 49 adult patients with focal epilepsy and 16 healthy subjects who underwent high-density-EEG and structural MRI. We estimated cortical activity using electric source analysis in 82 atlas-based cortical regions based on the individual MRI. We applied directed connectivity analysis (Partial Directed Coherence) on these sources and performed graph analysis: we computed the Global Efficiency on the whole brain and on each resting state network. We tested these features in different group of patients.

**Results:**

Compared to controls, efficiency was increased in both TLE and ETLE (p < 0.05). The somato-motor-network, the ventral-attention-network and the default-mode-network had a significantly increased efficiency (p < 0.05) in both TLE and ETLE as well as TLE with hippocampal sclerosis.

**Significance:**

During interictal scalp EEG epochs without IED, patients with focal epilepsy show brain functional connectivity alterations in the whole brain and in specific resting-state-networks. This higher integration reflects a chronic effect of pathological activity within these structures and complement previous work on altered information outflow. These findings could increase the diagnostic sensitivity of scalp EEG to identify epileptic activity in the absence of IED.

## Highlights

• Abnormal brain network is visible in absence of epileptic activity on hd-EEG.
• Higher efficiency in patients vs. controls in absence of epileptic activity on hd-EEG.
• Resting state networks are altered in epileptic patients.

## 1 Introduction

It is now well established that epilepsy is a network disease involving hyperexcitable neuronal networks (Laufs, 2012, Richardson, 2012) and it is therefore important to study the interactions occurring between different brain regions. Functional connectivity measures the statistical dependencies between different regions of the brain. Specific approaches, notably based on Granger-causality applied to high-density EEG, can reveal directional relationships between brain regions, i.e., the Granger-causal influence that one brain region exerts onto another. The presence of a common source influence the results and the interpretation must be carried out carefully, even if the correct flow directions are detected (Gourévitch et al., 2006, Seth et al., 2015). The complex networks obtained can be described and compared with graph-theory analysis (Bullmore and Sporns, 2009).

Resting-state EEG studies in epilepsy(Coito et al., 2019) have been used to characterise abnormal brain activity in the absence of epileptic activity detectable on scalp EEG and to distinguish patients with temporal lobe epilepsy (TLE) from healthy controls using regional cortical outflow (Verhoeven et al., 2018). During IED, dynamic brain network alterations seem related to interictal cognitive deficits (Coito et al., 2015) and surgical outcome (Carboni et al., 2019). However, IED-dependent changes in brain function are difficult to interpret without fundamental knowledge of functional differences in individuals with epilepsy at rest and limit the comparison with healthy controls, in whom IEDs are absent.

Different resting state networks have shown alterations in patients with epilepsy in fMRI studies (de Campos et al., 2016, Kay et al., 2013) as well as in MEG (Andrews-Hanna et al., 2014). These are the default mode network (Andrews-Hanna et al., 2014, Greicius et al., 2003, Raichle et al., 2001), the salience network and the fronto‐parietal attention network (Markett et al., 2014, Schmidt et al., 2016). EEG-based network analysis has shown additional directional information in resting state imaging (Coito et al., 2019) and could reveal important features of resting state networks (RSN) changes in epilepsy.

We sought to determine functional alterations during resting state in different subgroups of patients with focal epilepsy and in specific resting state networks, using high density EEG (hd-EEG). Here the network features at global level and at specific resting state level are described using Global Efficiency (GE) (Carboni et al., 2019, Rubinov and Sporns, 2010) to characterise the ability of the brain to integrate pathological information. This work enhances the network and subnetwork perspective on previous results that focused on the local scale in TLE, in terms of the summed outflow, to describe epileptic networks (Coito et al., 2019).

## 2 Methods

### 2.1 Patients

We selected 49 patients (median age 31 years old, range 14-60y, 24 females) from a total of 215 hd-EEG recordings acquired at the EEG and epilepsy unit of the Geneva University Hospital. These patients fulfilled the following criteria: (a) pharmacoresistant focal epilepsy with high-density (≥128 electrodes) EEG recording, (b) age older than 14 years, (c) no previous surgery. We excluded 166 patients due to: multifocal or generalised epilepsy (49), EEG technical problems (18), pre-operative MRI not suitable for head-model (16), paediatric (68), previous brain surgery (15).

This study was approved by the local ethics board.

Patients were divided into: (a) temporal lobe (37) and extratemporal lobe epilepsy (ETLE) (12), (b) lesional (35) and non-lesional (14). Supplementary Table 1 summarizes the patients’ clinical details.

### 2.2 Healthy controls

We recruited 16 healthy control subjects (median age 26 years old, range 9–54 y, 6 females).

### 2.3 EEG acquisition and pre-processing

High-density EEG recordings of healthy controls (128 or 256 electrodes, Electrical Geodesic system, sampling rate = 1000 Hz) were acquired at the University Hospital of Geneva. High-density EEG recordings of epileptic patients (128 or 256 electrodes, Electrical Geodesic Inc. system, sampling rate = 1000 Hz) were acquired in the context of pre-surgical evaluation at the University Hospital of Geneva. A board-certified EEG expert (PDS, BV, MS and SV) first visually identified and marked EEG epochs during wakefulness with eyes open without scalp EEG-visible epileptic activity. As simultaneous intracranial recordings were not available for these patients, we cannot exclude possible contamination of underlying epileptic activity not visible on the scalp EEG. We included 14/19 patients from previously published article from our group (Carboni et al., 2019).

**Fig. 1:**
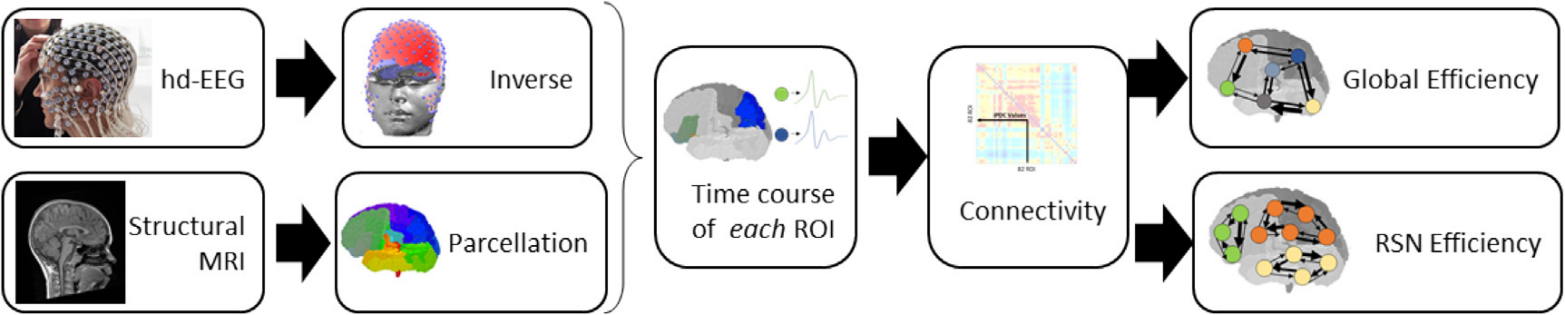
Summary of the analysis strategy: Hd-EEG and structural MRI (T1 or MPRAGE) were acquired. Around 5000 source-waveforms distributed equally in the grey matter were estimated by a distributed source localization algorithm (LAURA) from the resting state hd-EEG. The head model was based on the individual MRI and parcelled into 82 regions of interest (ROI). The activity in each ROI was summarised with a unique time-series through SVD. Connectivity matrices were estimated through iPDC. Efficiency was calculated for the entire brain and for the 7 resting state networks.

For both groups, we selected 45 epochs of 1-s EEG that were filtered in the interval [1–40] Hz with a 4th-order Butterworth filter to avoid phase-distortion and down-sampled at 250 Hz. EEG epochs containing artefacts were discarded after visual inspection, (Fig. 1 for methods steps).

### 2.4 MRI acquisition and pre-processing

For each patient and control subject, we created a realistic head model based on individual structural MRI image, either T1 or MPRAGE, recorded on a 3 T scanner (Siemens Prisma). Using Freesurfer v6.0.1 and the Connectome Mapper 3 open-source pre-processing software (Tourbier et al., 2019), we resampled each image to 1 mm^3^ isotropic resolution using cubic interpolation and we performed cortical and subcortical brain parcellation based on Desikan-Killiany (Desikan et al., 2006, Destrieux et al., 2010) anatomical atlas. This results in 82 parcels accounting for all grey matter structures, excluding brainstem and cerebellum. Each parcel was attributed to one of the 7 resting state networks of Yeo’s parcellation including the visual, somato-motor, dorsal attention, ventral attention, limbic, frontoparietal systems and default mode network (Yeo et al., 2011).

### 2.5 Source space solution

The computation of the individual head model, the linear distributed inverse solution, as well as the parcellation of the brain into 82 regions of interest (ROIs) was performed as described in our previous work(Carboni et al., 2019) as well as in the Appendix S1.

### 2.6 Connectivity estimation

As described in previous studies(Carboni et al., 2019, Coito et al., 2015, Rubega et al., 2019), we computed time-varying (tv) connectivity based on the Kalman filter approach for the estimation of high-dimensional tv-multivariate autoregressive (MVAR) models (Milde et al., 2010, Rubega et al., 2019). After the estimation of the MVAR coefficients, the connectivity matrices were estimated by applying the information Partial Directed Coherence (iPDC) in the frequency band [1–35] Hz with 0.5 Hz bins in order to have sufficient frequency resolution in the iPDC matrices. The magnitude of iPDC was considered for the subsequent analysis. Our epochs did not contain a specifically marked event to study the related brain dynamic and compare across “trials”/epochs. Nevertheless, we computed time-varying connectivity measure on the epochs and then averaged across the entire time series. Indeed, time-varying analysis is more suited for non-stationary signals, such as the spontaneous EEG.

Eventually, for each patient and control, we obtained a 4-dimensional matrix ([#ROIsx#ROIsxfrequencyxtime]) representing the directed information flow from one ROI to another for each frequency at each time point. We qualitatively inspected the data and the behaviour was consistent over frequencies and time, therefore the matrix was then averaged across frequencies [1–35 Hz] and time obtaining a 2-dimensional matrix ([#ROIsx#ROIs]).

### 2.7 Graph analysis

In order to extract information from these large connectivity matrices, the brain was represented as a weighted directed graph defined by a collection of nodes (ROIs) and edges (iPDC directed connections).

Subgraphs with all nodes in each separate resting state network were also built (Yeo et al., 2011). From these graphs, we calculated the global efficiency at the whole brain level within specific resting state networks (Latora and Marchiori, 2001, Rubinov and Sporns, 2010). These measures represent the ability of the network to combine specialized information from distributed brain regions [see Appendix for details].

### 2.8 Statistical analysis

#### 2.8.1 Whole brain efficiency

We constructed a whole brain graph with all the 82 ROIs as nodes and as edges the magnitude of the iPDC values. As described above, we computed the efficiency of the network. We used Bonferroni-corrected Mann-Whitney *U* test to compare (a) patients vs controls, (b) TLE patients vs controls, (c) ETLE patients vs controls, (d) TLE patients with MRI signs of hippocampal sclerosis vs controls, (e) non-lesional TLE patients vs controls, (f) non-lesional TLE&ETLE vs controls, (g) Right TLE vs Left TLE, (h) ILAE = 1–2 vs ILAE = 3–5. The Bonferroni correction was done over the number of comparions, i.e. 8. We furthermore computed the effect size for independent variables based on Cohen’s d: for d = 0.01: very small effect size, for d = 0.20: small effect size, for d = 0.50: medium effect size, for d = 0.80: large effect size, for d = 1.20: very large effect size and for d = 2.00: huge effect size. We computed the sensitivity, specificity, Positive and Negative Predictive Value(Lalkhen and McCluskey, 2008) for the whole brain efficiency of a) all patients, b) TLE, c) ETLE . The reference limit was settled at the 99th percentile of the healthy controls group. Finally we computed the correlation and the associated p-value between whole brain efficiency of the entire patient group and 3 clinical variables: Onset of Epilepsy, Duration of Epilepsy and ILAE classification.

#### 2.8.2 Resting state networks efficiency

We evaluated efficiency in each resting state network by building sub-graphs with nodes in each separate resting state network. We used a Bonferroni-corrected Mann-Whitney *U* test to compare the sub-networks’ efficiency (visual, somato-motor, dorsal attention, ventral attention, limbic, fronto-parietal systems and default mode network) in (a) patients vs controls, (b) TLE patients vs controls, (c) ETLE patients vs controls, (d) TLE patients with MRI signs of hippocampal sclerosis vs controls, (e) non-lesional TLE patients vs controls, (f) non-lesional TLE&ETLE vs controls, (g) Right TLE vs Left TLE. The Bonferroni correction was done over the number of RSN, i.e. 7. We furthermore computed the effect size as described above. Finally, we computed the correlation and the associated p-value between each RSN efficiency of the entire patient group and 3 clinical variables: Onset of Epilepsy, Duration of Epilepsy and ILAE classification.

## 3 Results

### 3.1 Whole brain efficiency

**Fig. 2:**
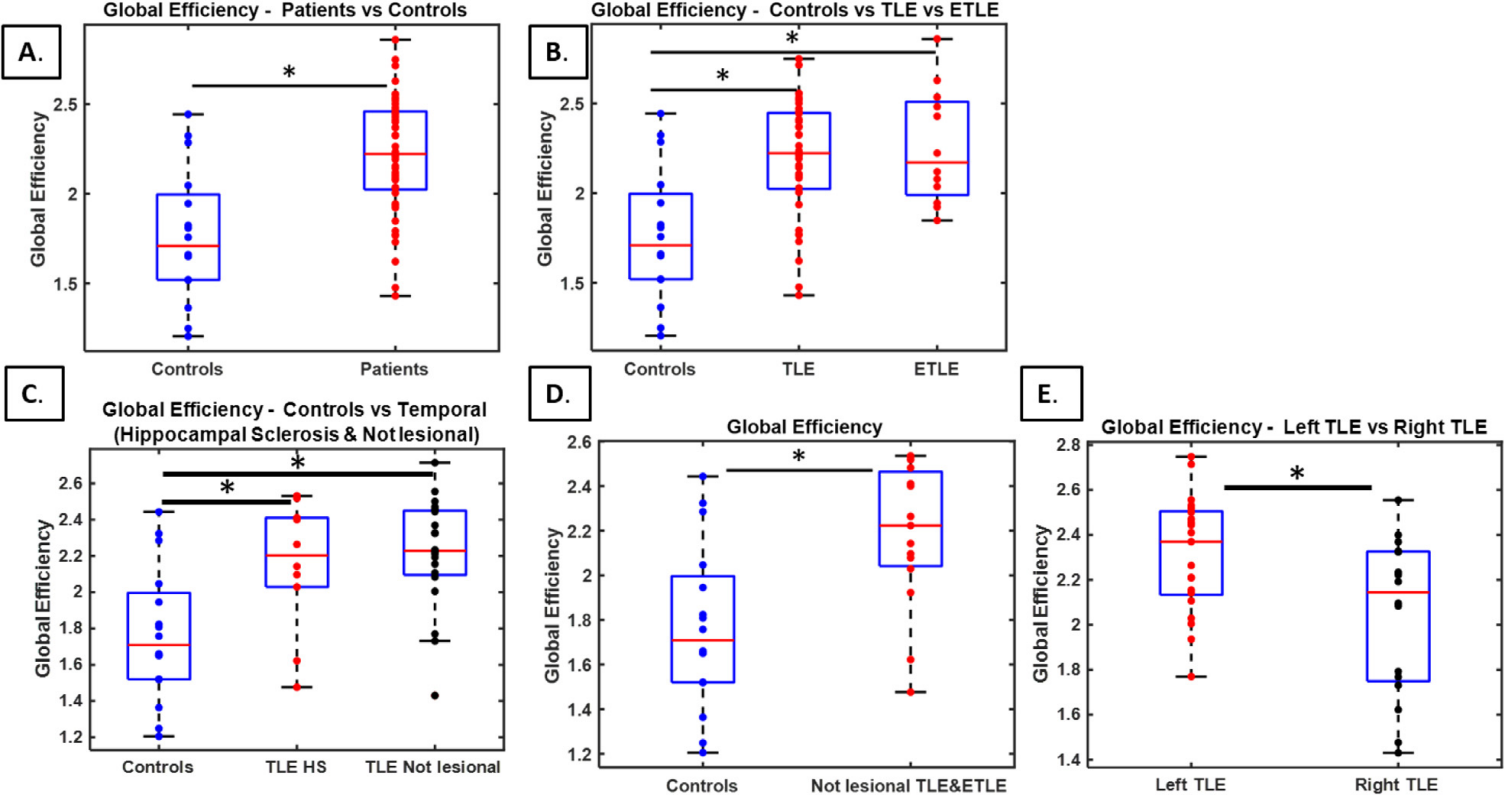
Global Efficiency: (a) controls vs patients (N = 49), (b) controls vs TLE (N = 37) vs ETLE (N = 12), (c) controls vs TLE with Hippocampal sclerosis (TLE HS, N = 20) vs TLE non-lesional (N = 9), (d) controls vs non-lesional (TLE-ETLE) (N = 15), (e) Left TLE (N = 14) vs Right TLE (N = 13). In each boxplot, the central line is the median value, the edges of the boxes are the 75th and the 25th percentiles.

For the entire network, we found an increased global efficiency in patients compared to controls (p < 0.001, effect size = 1.32) (Fig. 2 A). This was also found separately in patients with TLE (p = 0.003, effect size = 1.25) and in patients with ETLE compared to controls (p = 0.01, effect size = 1.40) (Fig. 2 B).

Furthermore, in subgroups of TLE, patients having hippocampal sclerosis (p = 0.005, effect size = 1.33) as well as those classified as non-lesional (p = 0.03, effect size = 1.04) showed an increased global efficiency compared with controls (Fig. 2 C). For the subgroup of all non-lesional TLE and ETLE we found an increased (p = 0.03, effect size = 1.19) global efficiency as compared with controls (Fig. 2 D). We found an increase (p = 0.02, effect size = 0.9) global Efficiency in Left TLE as compare to Right TLE (Fig. 2 E).

Finally, we further divided the subgroup of operated patients (N = 45) in good seizure outcome after surgery (ILAE = 1–2) and poor seizure outcome after surgery: we did not find any significant difference (p > 0.05, Supplementary Figure S1).

We found high specificity and positive predictive value for the three groups of patients but low sensitivity and low negative predictive value. (Table 1).

**Table 1:** Sensitivity, Specificity, Positive Predictive Value (Pos. Pred. Val.) and Negative Predictive Value (Neg. Pred. Val.) for all Patients, TLE and ETLE.

	Patients	TLE	ETLE
Sensitivity	28.50%	27%	33.30%
Specificity	93.70%	93.70%	93.70%
Pos. Pred. Val	93.30%	90.90%	80%
Neg. Pred. Val	30%	35%	65%

Finally, we did not find any significant correlation values (all p > 0.05) between the efficiency at whole brain level and any of the following clinical variables: Onset of Epilepsy, Duration of Epilepsy and ILAE (Supplementary Table 2): data for the different variables do not homogeneously cover the entire range of expected values, given the limited number of patients.

### 3.2 Resting state networks efficiency

**Fig. 3:**
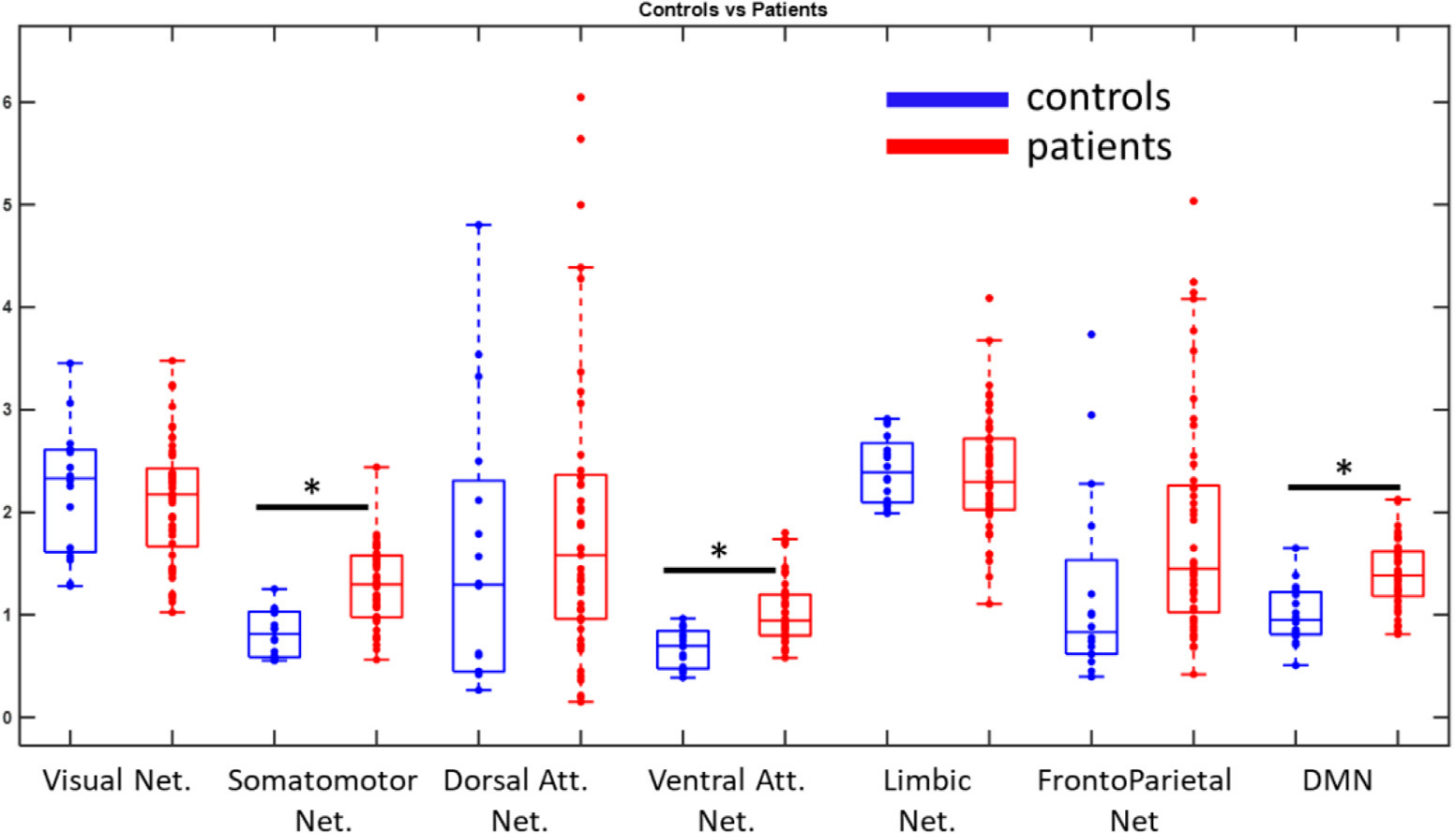
Global Efficiency in the different resting state networks for all patients (N = 49) (in red) and controls (in blue). For visualisation, two outliers in the Dorsal Attention Network Patients group have been removed. The central line is the median value, the edges of the boxes are the 75th and the 25th percentiles. (For interpretation of the references to colour in this figure legend, the reader is referred to the web version of this article.)

In all patients, we found a significant increase of the efficiency in the somato-motor network (p < 0.001, effect size = 1.36), in the ventral attention network (p < 0.001, effect size = 1.26) and in the default mode network (p = 0.001, effect size = 1.19) as compared to controls. (Fig. 3).

In all TLE and in TLE with hippocampal sclerosis (TLE-HS) we found a significant increase of the efficiency in the somato-motor (TLE: p < 0.001, effect size = 1.38, TLE-HS: p = 0.001, effect size = 1.49), ventral attention (TLE: p < 0.001, effect size = 1.27, TLE-HS: p = 0.004, effect size = 1.37), and default mode networks (TLE: p = 0.003, effect size = 1.20, TLE-HS p = 0.006, effect size = 1.35), as compared to healthy controls (Supplementary Figure S2). TLE patients without lesions showed no significant difference in any resting state network from healthy controls (p > 0.05) (Supplementary Figure S3). No significant difference was found between right and left TLE, in any resting state network (p > 0.05) (Supplementary Figure S3).

The subgroup of all non-lesional TLE and ETLE showed significant difference in the ventral attention network (non-lesional ETLE + TLE p < 0.001, effect size = 1.35) as compared to controls (Supplementary Figure S4).

Other lesions could not be grouped in sufficiently large groups for analysis.

In ETLE patients we found a significant increase of the efficiency in the somato-motor network (p = 0.01, effect size = 1.55), in the ventral attention network (p = 0.01, effect size = 1.45) and in the default mode network (p = 0.03, effect size = 1.21), as compared to healthy controls, similarly to the temporal lobe patients (Supplementary Figure S5). Given the size of the ETLE sample, no further subgroup analysis was performed.

Finally, we did not find significant correlation values (p > 0.05) between the efficiency at in any RSN and any clinical variable such as the Onset of Epilepsy, Duration of Epilepsy and ILAE: data for the different variables do not span the entire range give the limited number of patients.

## 4 Discussion

Our study investigated the dynamic resting state connectivity patterns in focal epilepsy, in order to improve our understanding of the complex interplay between pathological areas and whole brain networks. We measured network efficiency at the global brain level and in specific resting state networks to test for alterations in network integration related to focal epilepsy.

For both TLE and ETLE, we found an increased global efficiency compared to healthy controls. Increased efficiency reflects higher integration of different brain area and can be interpreted as a more extensive pathological (epileptic) network within the brain. In our previous work, increased efficiency was found during IED of patients with poor versus good outcome of epilepsy surgery (Carboni et al., 2019). In the current study, such increased integration appears as a fundamental aspect of brain network reorganisation in patients with epilepsy, even in the absence of EEG-visible epileptic activity. The finding of increased network efficiency during IEDs and in their absence is supported by simultaneous EEG-fMRI studies that showed similar epileptic network patterns during IEDs and in their absence (Iannotti et al., 2016). In other epileptic conditions, increased efficiency in infants with tuberous sclerosis was found to be predictive of the subsequent occurrence of epileptic spasms, therefore also suggesting more widespread epileptic networks (Davis et al., 2019). In Benign Epilepsy with Centro-Temporal Spikes (BECTS) results are more difficult to interpret, due to heterogeneity of EEG analysis strategies (Adebimpe et al., 2016, Ji et al., 2017). Further, increased connectivity patterns independent of focal IEDs were measured by intracranial EEG analysis (Bettus et al., 2009).

These combined findings strengthen the role of network efficiency and integration measures as markers of hyperexcitable pathologic activity associated with epilepsy. Beyond the transient impact of IED on brain networks, these IED-independent alterations offer a promising approach to study interictal alterations.

In our study we show large effect size, allowing the hypothesis that GE at both whole brain scale and RSN scale could be used as meaningful feature in diagnostic algorithms. In recent years, some algorithms have already reported high sensitivity and specificity with the use of classifiers based on machine learning (Verhoeven et al., 2018). Our very high specificity and positive predictive value for the three groups of patients suggest that, if present, increased efficiency could have a confirmatory diagnostic role. The lack of relevant correlation with some clinical variables including outcome after surgery could be related to the patient’s population heterogeneity.

Further studies that consider connectivity measures together with other clinical/neuropsychological variable are needed to explain changes in the EEG connectivity and its added value at individual level for diagnosis or monitoring of disease activity in the absence of IED on scalp EEG. The situation of non-lesional epilepsies is particularly relevant for diagnostic purposes. In the absence of lesion and sometimes visually normal EEG recordings, additional biomarkers are needed.

### 4.1 Whole brain efficiency in TLE

We consistently found increased efficiency in TLE compared to controls. In our previous connectivity studies based on high-density EEG, we reported other network characteristics in TLE, such as summed outflow (main drivers)(Coito et al., 2016) and clustering coefficient (segregation)(Coito et al., 2019). Here we focused on efficiency as a marker of network integration, considering that such measure could represent a reliable marker of the propagation of epileptic activity and therefore of the extension of the epileptic network. Since the activity of an entire network is dependent on the functional interaction between its nodes, even small changes in connectivity may cause dysfunction in global brain networks (Cataldi et al., 2013). In this work we extended previous local results based on the driving importance of each node (summed outflow) (Coito et al., 2016) by adding insight on the information transfer between nodes (edge/global network information) on a resting state network scale(Schaefer et al., 2018, Yeo et al., 2011) and a whole functional connectivity networks(Ridley et al., 2015) scale. Concordant results were obtained in a resting state fMRI study where increased efficiency of the thalamus was correlated with poor post-surgical outcome, again suggesting widespread propagation of epileptic activity (He et al., 2017).

### 4.2 Whole brain efficiency in left and right TLE

The global efficiency was significantly higher in left TLE compared to right TLE. Comparison with our previous TLE connectivity studies based on high-density EEG is difficult, as we previously reported other network characteristics, such as summed outflow (main drivers) (Coito et al., 2016)and clustering coefficient (segregation)(Coito et al., 2019). Here we focused on efficiency as a marker of network integration and of the propagation of epileptic activity in the epileptic network. In the current approach we added a perspective not only at the whole-brain networks but also in specific resting state networks (Schaefer et al., 2018, Yeo et al., 2011) (Ridley et al., 2015).

Despite widespread reported connectivity alterations in both patients’ groups, more bilateral abnormalities have been described in right vs left TLE (Coito et al., 2015, de Campos et al., 2016). Structural connectivity studies show greater and more diffuse changes in left TLE, compared to primarily ipsilateral changes in right TLE (Ahmadi et al., 2009).

The comparison between studies is not trivial, due to different functional connectivity and network analyses. Here, we measured the contribution of all the brain regions to the efficiency of the network and the results cannot be compared to the asymmetries of outflow from a few selected high drivers. The resting state networks were considered as bilateral and symmetrical sub-network preventing lateralisation analysis. Lateralization effects of ipsilateral temporal epileptic regions were therefore potentially washed out by the contribution of all other regions. Furthermore, in temporal lobe disorders, a range of imaging data supports an association of left-lateralised epilepsies with a greater burden of changes in connectivity (Ridley et al., 2015).

### 4.3 Whole brain efficiency in ETLE

Patients with ETLE showed alterations in the same RSN as temporal lobe patients. Earlier simultaneous EEG-fMRI studies suggested distinct spatial patterns for FLE and TLE (Fahoum et al., 2012). Our ETLE patients were heterogeneous in their expression, aetiology, semiology and prognosis even if gathered in the same group (Berg et al., 2010). The limited number of ETLE patients precluded further subgroup analysis, notably FLE. The brain parcellation into regions that did only partly overlap with specific RSN may also have reduced the specificity of our findings regarding selected patient groups.

### 4.4 Resting state network efficiency in TLE and ETLE

Despite an overall higher global efficiency in TLE with and without hippocampal sclerosis vs controls, the resting state networks in patients with HS appear more severely altered than in non-lesional cases, with a specific alteration in DMN, somato-motor and ventral attention. In our previous TLE study involving a majority of HS (24/40), connectivity alterations were found in regions overlapping with the DMN, but connectivity changes inside the DMN (i.e. between DMN regions) had not been investigated yet(Coito et al., 2019). A strong correspondence between hippocampal activity and parts of the DMN has been previously shown (Laufs et al., 2007). We cannot determine if the group difference in our study reflects a deeper or more syndrome-specific medial temporal dysfunction in TLE with HS or whether non-lesional TLE were less localised in the medial temporal structures. Furthermore, no differences were found in left vs right TLE. In ETLE widespread alterations were found in the different resting state networks despite the variable localisation of the epileptic focus and in the absence of IED.

Alterations in the somatomotor network in focal epilepsy, particularly TLE, could be related to the frequent abnormal findings in precentral cortex (part of the somatomotor network) in functional and structural connectivity studies (Lemkaddem et al., 2014, Trimmel et al., 2018), as well as morphometric ones (Garcia et al., 2017). Impairment of the ventral attention network in TLE during an “oddball paradigm’’ has been described with high-density EEG(Bocquillon et al., 2009) and linked with reduced attentional performances(Fleck et al., 2002). These differences, found also during task-free condition, strengthen the view that TLE chronically modifies functional brain networks (Cataldi et al., 2013).

In non-lesional cases, we found abnormalities in the ventral attention network but results in this subgroup could have been biased by a mixed effect of both TLE and ETLE, with ETLE driving this result. The clinical usefulness of this findings needs to be specifically addressed, with balanced numbers of TLE and ETLE. Interestingly, the other networks were not altered, suggesting a less widespread involvement of epileptic activity in non lesional epilepsy.

### 4.5 Methodological considerations

As in our study, most of the previous graph theory studies using fMRI in temporal lobe epilepsy have applied anatomical parcellation to functional maps and compared differences in temporal lobe patients sub-groups in comparison to controls (Bettus et al., 2009, Wang et al., 2009). Moreover, networks obtained from MEG and EEG recordings are similar to the fMRI RSNs (Britz et al., 2010, Brookes et al., 2011, Chen et al., 2013, Liu et al., 2017). Therefore, selectively mapping alterations of these functional interactions may improve the identification of changes related to neurological disorders. In our regrouping of ROI into RSN, the resting state network did not always follow the anatomical border so that the attribution of one brain area to one resting state network was based on the largest overlap. One of the consequences was that there were few regions attributed to the limbic network. This could explain the lack of difference in the limbic network measures, notably in TLE due to insufficient statistical power. The low contribution from the medial temporal regions to the EEG signal may have added to this problem. The atlases used in EEG source studies have been primarily developed for fMRI analysis and region shape and extent may not be adequately summarized by one single source signal. New atlas parcellation options, including cortical regions better represented by a single EEG source signal and reflecting the spatial organization of resting state networks, would enhance the relevance of connectivity analysis based on ESI.

The sample size of patients with extra-temporal epilepsy was limited, thus preventing further analysis of lateralization and localization of network alterations in this population. Also, our study was underpowered to investigate correlations between connectivity measures and clinical variables (age, disease duration, seizure semiology, drug load.

## CRediT authorship contribution statement

**Margherita Carboni:** Conceptualization, Conceptualization, Software, Validation, Formal analysis, Data curation, Visualization. **Pia De Stefano:** Conceptualization, Data curation, Visualization. **Bernd J. Vorderwülbecke:** Data curation, Writing - review & editing. **Sebastien Tourbier:** Software, Validation, Writing - review & editing. **Emeline Mullier:** Software, Validation. **Maria Rubega:** Methodology, Software. **Shahan Momjian:** Resources, Writing - review & editing. **Karl Schaller:** Writing - review & editing. **Patric Hagmann:** Writing - review & editing, Funding acquisition. **Margitta Seeck:** Supervision, Resources, Writing - review & editing, Funding acquisition. **Christoph M. Michel:** Methodology, Writing - review & editing, Supervision, Funding acquisition. **Pieter van Mierlo:** Conceptualization, Methodology, Software, Formal analysis, Funding acquisition. **Serge Vulliemoz:** Conceptualization, Methodology, Investigation, Resources, Supervision, Project administration, Funding acquisition.

## Declaration of Competing Interest

The authors declare that they have no known competing financial interests or personal relationships that could have appeared to influence the work reported in this paper.

## Appendix 1

### Selection of frequency for connectivity analysis

We filtered the EEG data between 1 and 40 Hz, the high pass was chosen in order to remove possible slow drifts in the recordings. The duration of the epochs selected, i.e. 1 sec., is typical of other related studies. Formally, a longer window would be needed to precisely estimate the connectivity in the lowest part of the selected frequency band (1–2 Hz). However, when adding/removing the lowest frequency bins, we did not experience a notable effect of on the broadband connectivity estimation so that the impact of slow frequency bins is considered to be low.

## Inverse solution

For the forward model, we used a simplified realistic head model with consideration of skull thickness (Locally Spherical Model with Anatomical Constraints (LSMAC))(Michel and Brunet, 2019) and a grid of around 5000 sources (solution points), distributed equally in the grey matter. Both the lead-field matrix and the inverse matrix were computed using the freely available software Cartool (Michel and Brunet, 2019) and the inverse solution performed with LAURA (Local AUtoRegressive Average). Epochs were transformed into source-waveforms at every solution point contained in the 82 regions of interest (ROIs) defined above. As the representative time-series for each region, we considered the first singular vector computed by a singular-value decomposition of all the 3D source-waveform (dipoles) in the same ROI(Rubega et al., 2019).

## Network measures

*Global efficiency* evaluates the ability of the brain to rapidly combine specialized information from distributed brain regions(Latora and Marchiori, 2001). The global efficiency measures the inverse of the shortest path length. Structural networks usually are similarly organized and share a high global efficiency whereas functional networks have weaker connections between modules and consequently a weaker global efficiency(Honey et al., 2007).

(2) $$\vec{E}=\frac{1}{n}\sum_{i\in N}\frac{{\sum_{j\in N,j\neq i}\left({\vec{d}}_{\mathrm{ij}}\right)}^{-1}}{n-1}$$

Ethical Publication Statement

We confirm that we have read the Journal’s position on issues involved in ethical publication and affirm that this report is consistent with those guidelines.

Due to data protection reasons, data cannot be made publicly available. However, anonymized grouped data will be made available upon reasonable request to qualified investigators.
